# MLVA Genotyping of *Brucella melitensis and Brucella abortus* Isolates from Different Animal Species and Humans and Identification of *Brucella suis* Vaccine Strain S2 from Cattle in China

**DOI:** 10.1371/journal.pone.0076332

**Published:** 2013-10-04

**Authors:** Hai Jiang, Heng Wang, Liqing Xu, Guiying Hu, Junying Ma, Pei Xiao, Weixing Fan, Dongdong Di, Guozhong Tian, Mengguang Fan, Jingchuan Mi, Ruiping Yu, Litao Song, Hongyan Zhao, Dongri Piao, Buyun Cui

**Affiliations:** 1 State Key Laboratory for Infectious Disease Prevention and Control, Collaborative Innovation Center for Diagnosis and Treatment of Infectious Diseases, National Institute for Communicable Disease Control and Prevention, Chinese Centre for Disease Control and Prevention, Beijing, China; 2 Laboratory of Endemic and Parasitic Diseases Control and Prevention, Hangzhou Center for Disease Control and Prevention, Hangzhou, China; 3 Laboratory of Brucellosis, Qinghai Institute for Endemic Disease Prevention and Control, Xining, China; 4 Laboratory of Zoonoses, China Animal Health and Epidemiology Center, MOA, Qingdao, China; 5 Department of Brucellosis, Inner Mongolia Center of Endemic Disease Control and Research, Huhhot, China; Institut National de la Recherche Agronomique, France

## Abstract

In China, brucellosis is an endemic disease and the main sources of brucellosis in animals and humans are infected sheep, cattle and swine. *Brucella melitensis* (biovars 1 and 3) is the predominant species, associated with sporadic cases and outbreak in humans. Isolates of *B. abortus*, primarily biovars 1 and 3, and *B. suis* biovars 1 and 3 are also associated with sporadic human brucellosis. In this study, the genetic profiles of *B. melitensis* and *B. abortus* isolates from humans and animals were analyzed and compared by multi-locus variable-number tandem-repeat analysis (MLVA). Among the *B. melitensis* isolates, the majority (74/82) belonged to MLVA8 genotype 42, clustering in the ‘East Mediterranean’ group. Two *B. melitensis* biovar 1 genotype 47 isolates, belonging to the ‘Americas’ group, were recovered; both were from the Himalayan blue sheep (*Pseudois nayaur*, a wild animal). The majority of *B. abortus* isolates (51/70) were biovar 3, genotype 36. Ten *B. suis* biovar 1 field isolates, including seven outbreak isolates recovered from a cattle farm in Inner Mongolia, were genetically indistinguishable from the vaccine strain S2, based on MLVA cluster analysis. MLVA analysis provided important information for epidemiological trace-back. To the best of our knowledge, this is the first report to associate *Brucella* cross-infection with the vaccine strain S2 based on molecular comparison of recovered isolates to the vaccine strain. MLVA typing could be an essential assay to improve brucellosis surveillance and control programs.

## Introduction

Brucellosis, recognized as a zoonotic disease of global importance, is caused by bacteria of the genus *Brucella*, which currently encompasses ten recognized species [1-3]. *B. melitensis* predominantly infects sheep and goats, *B. abortus* infects cattle, and *B. suis* infects swine and a range of wild animals. Cross-infection of other mammalian species, including humans, may occur [4]. Brucellosis is prevalent in China, especially in northern China, where people are economically dependent on ruminant livestock. *B. melitensis* has been the predominant species associated with human outbreaks and sporadic cases in China; *B. abortus* and *B. suis* are also associated with sporadic epidemics [5]. Based on analysis of epidemiological data in the 1990s, the incidence of animal brucellosis is stable and relatively low, whereas the incidence of human brucellosis during this time increased. The reason was probably due to the improved surveillance or public awareness [5]. Since 2008, 21 sentinel surveillance sites for animal and human brucellosis were established in 19 provinces nationwide. During this 5 years period, a number of animal and human isolates have been collected. To compare the epidemiological relationships of *Brucella* isolates recovered from different sources, a multi-locus variable-number tandem-repeat analysis (MLVA) assay was used [6]. In China, brucellosis is an endemic disease where *B. melitensis* biovars 1 and 3 and *B. abortus* biovars 1 and 3 are the prevailing species. *B. suis* isolates are scarce [5]. MLVA genotyping of Chinese human *B. melitensis* isolates and *B. suis* biovar 3 isolates have been reported previously by our team [7]. However, none of these studies included animal isolates. Thus, the primary aim of this study was to achieve globally *Brucella* isolates baseline genotyping data and assess the diversity among strains for epidemiological purposes in human and animal brucellosis in China.

In animals, *Brucella* vaccination is widely used for the prevention and control of brucellosis. Unique live, attenuated *Brucella* vaccines have been used depending on the preferred host, such as *B. abortus* S19 for cattle and *B. melitensis* Rev. 1 for sheep and goats [8,9]. The two vaccines, when administered correctly, can protect live-stock from brucellosis but can still cause abortions when administered at the wrong time [10-13]. Furthermore, while the vaccines are considered sufficiently attenuated for animal use, they may still be pathogenic to humans. There are documented cases showing the pathogenic nature of strains Rev. 1 and S19 in humans [14]. Thus, for the effective monitoring of both brucellosis control programs and human disease it is important to have reliable tests to differentiate vaccine and field strains. Many molecular approaches have been developed to detect vaccine strains [15-18]. The live attenuated strain *Brucella suis* S2 was isolated from swine fetus by the China Institute of Veterinary Drug Control (IVDC) in 1952. It has been passaged *in vitro* for more than 100 generations during the last 2 decades, and is the most widely used animal vaccine against brucellosis in China. The vaccine is administered through drinking water; a dose of 10 billion bacterial gives 2-3 years of protection [5]. To date, no specific molecular markers have yet been identified for this vaccine strain.

Species identification and subtyping of *Brucella* isolates are very important for epidemiologic surveillance and investigation of outbreaks in brucellosis endemic regions [19,20]. Previous studies have confirmed that MLVA is a useful tool for identifying and genotyping *Brucella* isolates and the resultant data can be used for epidemiological trace-back investigations [21-24]. Recently, a MLVA assay was used to assess the genetic stability of the *B. melitensis* Rev. 1 vaccine strain [25]. The lack of specific molecular markers has hampered attempts to distinguish the S2 vaccine strain from field isolates. In this report, we also present the results of an investigation employing the *Brucella* MLVA assay to specifically address the identification of the S2 vaccine strain in animals in China.

## Results

### MLVA 16 typing and clustering of *B. melitensis* and *B. abortus* population

**Figure 1:**
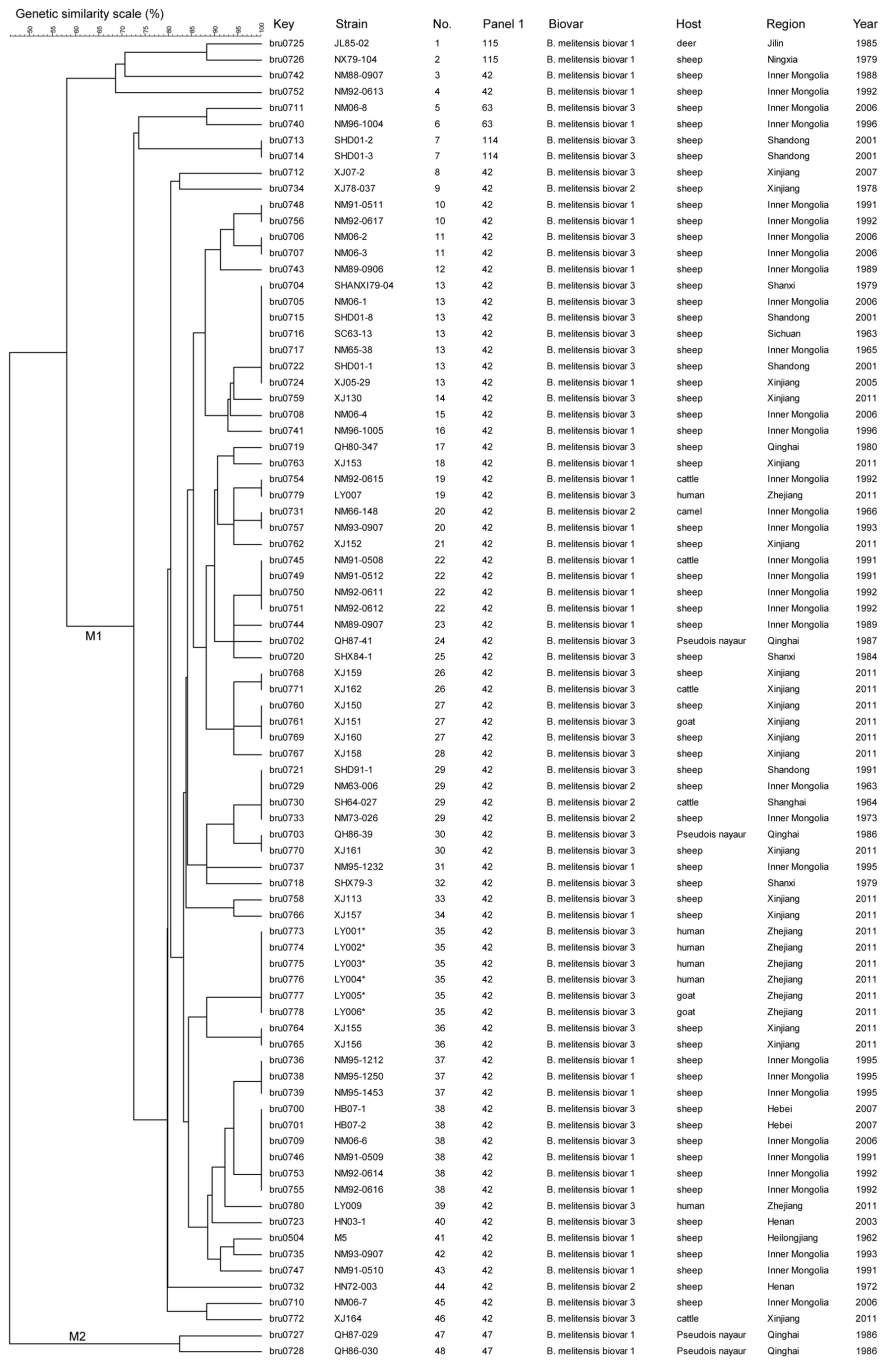
Dendrogram based on the MLVA-16 genotyping assay showing relationships of the 82 *B. melitensis* strains in this study. In the columns, the following data for isolates are indicated: Key: serial number for the isolate in the Brucella2012MLVA database (Key), strain name in the laboratory (strain), genotype numbering (No.), Panel1 genotypes corresponding to each isolate in the Brucella2012, geographic region (Region), year of isolation(Year). Outbreak isolates recovered from Longyou country are marked with an asterisk.

Using the complete MLVA-16 assay including panel 1, 2A and 2B loci, 82 *B. melitensis* isolates clustered into 48 genotypes with a genetic similarity coefficient ranging from 58.14 to 100% (Figure 1). Two clearly distinct clusters could be defined, M1 and M2 at 58.14% similarity. By panel 1, the *B. melitensis* population clustered into five MLVA8 genotypes; three previously described and two novel. Two of the three known genotypes(genotype 42, 74 isolates and genotype 63, 2 isolates) are part of the ‘East Mediterranean’ group. The third previously recognized genotype (genotype 47, 2 isolates) is a member of the ‘Americas’ group. The two novel genotypes had a panel 1 MLVA profile of 1-5-3-13-2-1-3-2 and 1-4-3-13-2-2-3-2, and have been assigned numbers 114 and 115 in MLVAbank (http://mlva.u-psud.fr/)... Both represent single-locus variants (SLV) of MLVA8 genotype 42 (1-5-3-13-2-2-3-2). The most discriminatory panel 2B markers were bruce04, bruce16 and bruce30, with a diversity index of > 0.7 harboring 6, 9 and 7 alleles respectively. Considering all three MLVA panels, the number of strains sharing the identical MLVA16 genotype ranged from two to seven. Outbreak isolates recovered from Longyou country are marked with an asterisk (Figure 1). The main characteristics of the 16 loci in the *B. melitensis* strains are shown in Table 1.

**Table 1:** Allelic types and Hunter and Gaston Diversity index (HGDI) of *B. melitensis* and *B. abortus* isolates for 16 loci in this study.

	Locus	*B. melitensis*				*B. abortus*			
		ATs^a^	TRs^b^	HGDI^c^	CI^d^	ATs^a^	TRs^b^	HGDI^c^	CI^d^
Panel 1	Bruce 06	2	1,3	0.048	0.000-0.113	1	4	0	0
	Bruce 08	2	4-5	0.094	0.008-0.180	1	5	0	0
	Bruce11	2	2-3	0.048	0.000-0.113	3	2-4	0.185	0.067-0.303
	Bruce 12	1	13	0	0	1	12	0	0
	Bruce 42	2	2,4	0.048	0.000-0.113	1	2	0	0
	Bruce 43	3	1-3	0.014	0.004-0.224	1	2	0	0
	Bruce 45	1	3	0	0	1	3	0	0
	Bruce 55	2	2-3	0.048	0.000-0.113	3	1-3	0.395	0.271-0.519
Panel 2A	Bruce 18	2	4,6	0.007	0.000-0.148	3	6-8	0.136	0.028-0.245
	Bruce 19	4	20-23	0.223	0.106-0.348	2	17,21	0.029	0.027-0.084
	Bruce 21	1	8	0	0	2	8-9	0.029	0.027-0.084
Panel 2B	Bruce 04	6	3-8	0.732	0.690-0.779	3	4-6	0.528	0.423-0.633
	Bruce 07	4	4-8	0.118	0.022-0.215	4	4-7	0.562	0.448-0.676
	Bruce 09	6	1,3-4,6-8	0.186	0.007-0.301	10	3-12	0.842	0.809-0.875
	Bruce 16	9	2-10	0.826	0.786-0.866	2	3-4	0.183	0.068-0.297
	Bruce 30	7	3-8,10	0.749	0.678-0.820	2	3,5	0.205	0.008-0.323

a Allelic types

b The total number of repeat units at each locus was determined by the correlation with the amplicon size according to previously published reports (Materials and Methods)

c Hunter and Gaston index

d Precision of the diversity index, expressed as 95% upper and lower boundaries

**Figure 2:**
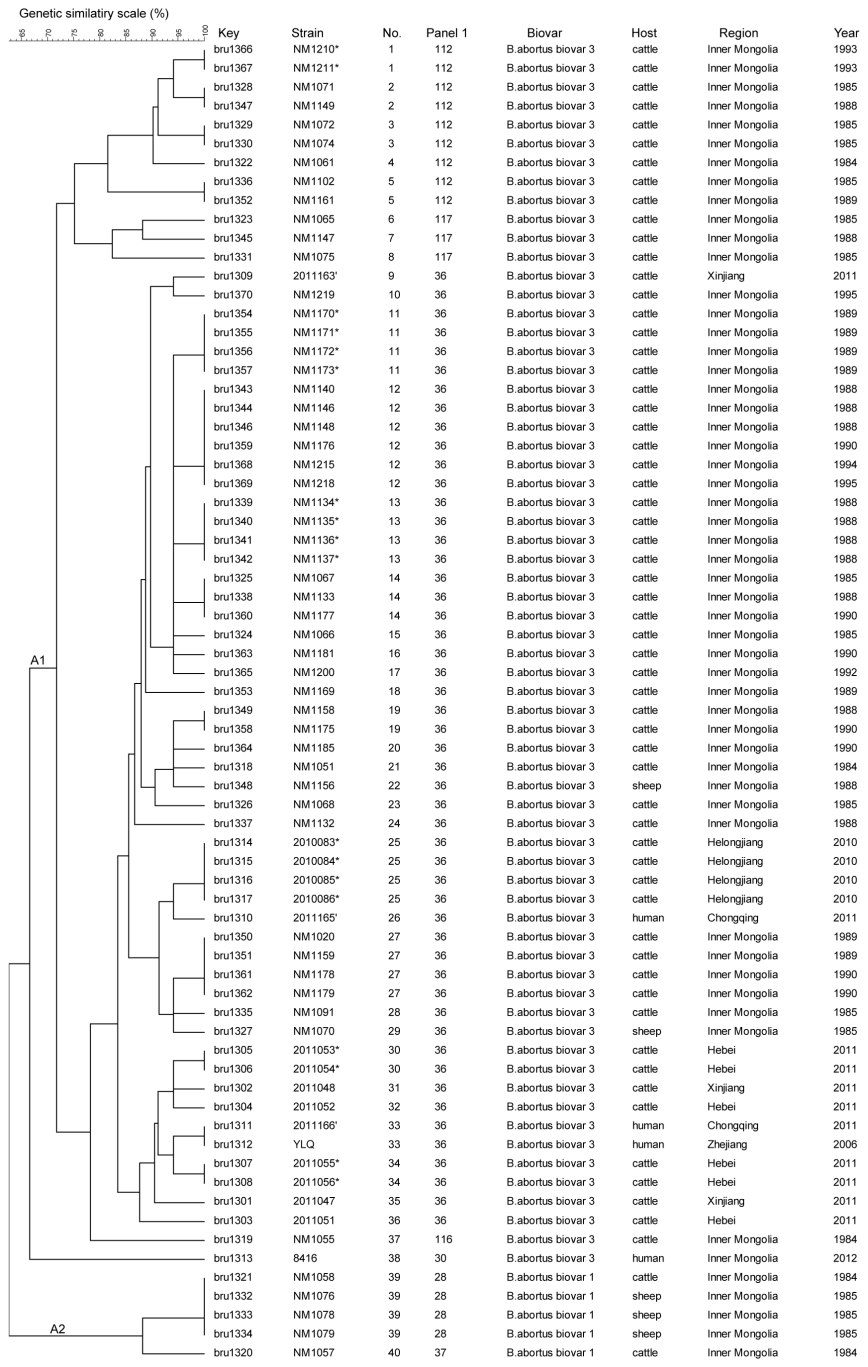
Dendrogram based on the MLVA-16 genotyping assay showing relationships of the 70 *B. abortus* strains in this study. In the columns, the following data for isolates are indicated: Key: serial number for the isolate in the Brucella2012MLVA database (Key), strain name in the laboratory (strain), genotype numbering (No.), Panel1 genotypes corresponding to each isolate in the Brucella2012, geographic region (Region), year of isolation (Year). Clusters of outbreak isolates are marked with an asterisk.

The 70 *B. abortus* isolates clustered into 40 different genotypes with a genetic similarity coefficient ranging from 66.57 to 100% (Figure 2). The isolates were divided into two clusters, A1 and A2 respectively at 66.57% similarity. The vast majority (65/70) of cluster A1 isolates belonged to biovar 3 while cluster A2 consisted of all isolates from biovar 1. The panel 1 marker bruce11 differentiated *B. abortus* biovar 1 and biovar 3 isolates (four and three repeats, respectively). However, only four known panel 1 (MLVA8) genotypes (28, 30, 36 and 37) were detected. The most discriminatory marker was panel 2B marker bruce09, with a diversity index of >0.8, consisting of 10 alleles. Three new panel 1 genotypes were detected. These were recorded as 4-5-3-12-2-2-3-3, 4-5-2-12-2-2-3-1 and 4-5-3-12-2-2-3-2, which are recently numbered 112, 116 and 117 in MLVAbank. Each genotype was a SLV to MLVA8 genotype 36 (4-5-3-12-2-2-3-1). The number of strains sharing the same MLVA16 genotype ranged from two to six. Outbreak isolates are marked with an asterisk (Figure 2). The main characteristics of the 16 loci in the *B. abortus* strains are shown in Table 1.

### Epidemiological relationship between human and animal *B. melitensis* isolates from Longyou, Zhejiang, 2011

Among 15 homogenous clusters, one cluster consisted of six outbreak related isolates (bru0773-bru0778). The MLVA-16 profile is 1-5-3-13-2-2-3-2-4-20-8-6-4-3-4-4. The genotype was a DLV to the two Xinjiang isolates bru0764 and bru0765 (1-5-3-13-2-2-3-2-4-20-8-5-4-3-3-4). These isolates were recovered from Longyou county, Zhejiang province, in 2011. All of the isolates were collected within one week from late July to early August. Four patients (bru0773-bru0776) had identified occupational exposure risks history and experienced fever, debility and joint pains. Two isolates, bru0774 and bru0776, were obtained from a married couple who had contact with sheep fetuses and placenta from a farm. Isolates bru0777 and bru0778 were recovered from goats in the outbreak region. The six strains from the recent outbreak shared the same genotypes, clearly showing the epidemiological relation between isolates from animal and human origin.

### Genetic characteristics of *B. abortus* isolates from human and animal

The majority of *B. abortus* isolates were recovered from cattle from Inner Mongolia, an area in China of known endemicity. The most often recovered genotype 36 was distributed in animals and humans. The human analysis was limited to four isolates. The two Sichuan isolates (bru1310 and bru1311) were collected from asymptomatic infections in geographically separate areas and they differed only by the loss of one repeat unit at locus bruce07. Two additional isolates were recovered from patients who worked on a livestock farm that had reported bovine brucellosis. Among 15 homogenous clusters, 6 clusters consisted of epidemiologically related isolates exclusively from the same province and the same time, represented an outbreak respectively. Among four human isolates, two isolates (bru1311 and bru1312) had the identical MLVA pattern with no apparent epidemiological link.

### Epidemiological trace-back of the vaccine-related *B. suis* biovar 1 strains infection in cattle

**Figure 3:**
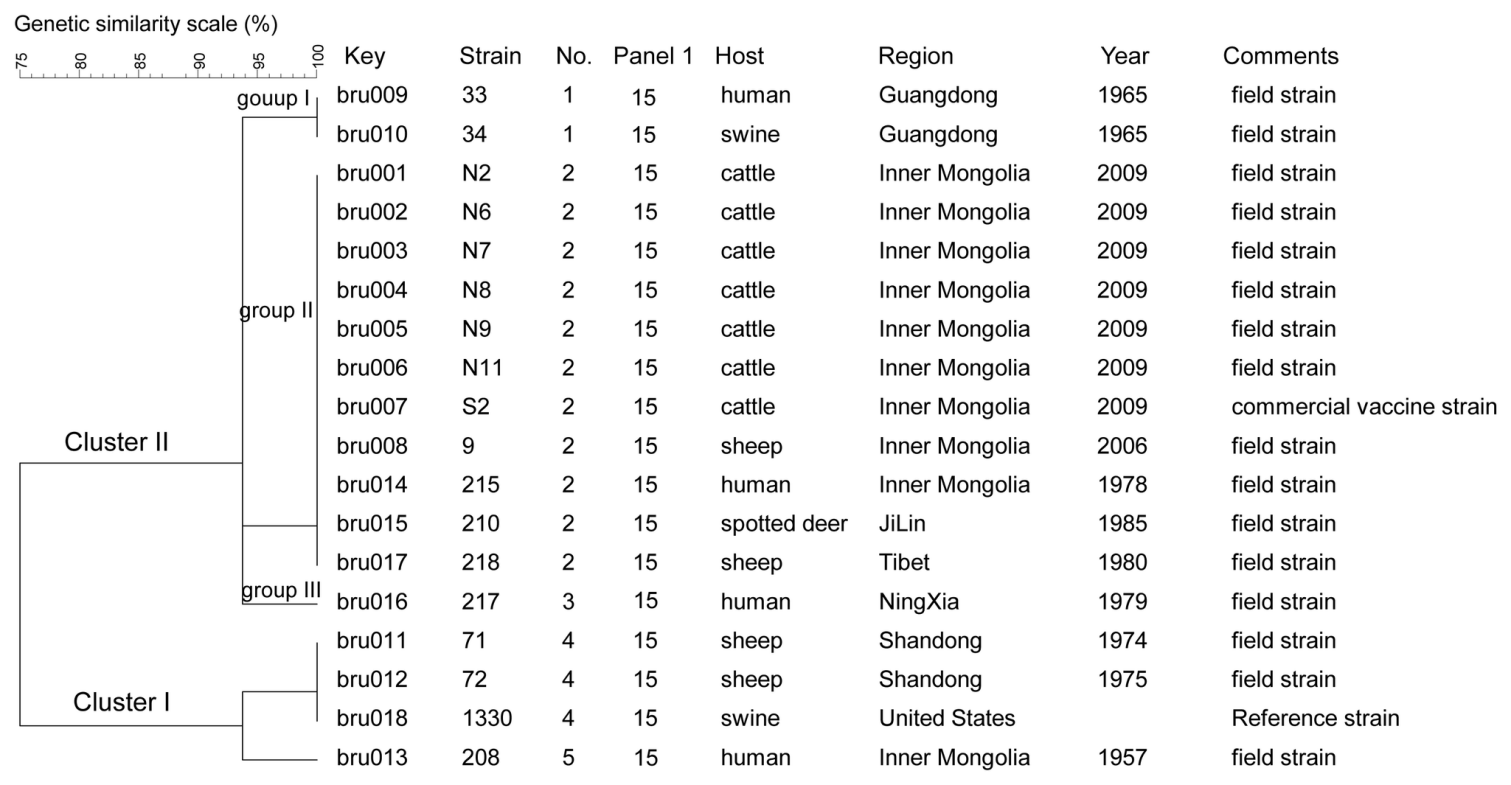
Dendrogram based on the MLVA-16 genotyping assay showing relationships of the 18 *B. suis* biovar 1 strains in this study. In the columns, the following data for isolates are indicated: Key: serial number for the isolate in the Brucella2012MLVA database (Key), strain name in the laboratory (strain), genotype numbering (No.), Panel1 genotypes corresponding to each isolate in the Brucella2012, geographic region (Region), year of isolation (Year).

A genetic passage experiment was performed to assess the stability of the S2 vaccine strain genetic markers over time. No changes in the MLVA-16 genotype (2-3-6-10-4-1-5-2-4-19-9-5-5-8-6-3) were observed in the vaccine strain S2 even after 40 passages *in vitro*. MLVA profiles obtained from 16 *B. suis* biovar 1 field isolates were compared using the web-based Brucella2012 MLVA database (http://mlva.u-psud.fr/). According to the database, a strain “BCCN87-67a”with an identical MLVA16 genotype has already been described. Interestingly, this strain is indicated as originating from China. MLVA-8 and MLVA-11 did not reveal any diversity (MLVA-8 profile: 2-3-6-10-4-1-5-2; MLVA-11 profile: 4-19-9). However, the most discriminatory VNTR marker was panel 2B bruce09 with a diversity index of 61.44% and consisting of five alleles. MLVA-16 analysis separate the 18 *B. suis* biovar 1 isolates, including seven isolates recovered from an outbreak at a cattle farm in Inner Mongolia and isolates from five Chinese Provinces [Shandong (in 1974 and 1975), Guangdong (1965), Ningxia (1979), Jilin (1985), and Tibet (1980)], into two clusters at 75% similarity (Figure 3). Cluster I consisted of four isolates, while cluster II had 14 isolates that grouped into 3 unique branches, Groups I, II, and III. The only difference observed between isolates was at the bruce09 locus. The largest branch was composed of 11 isolates; all had an indistinguishable MLVA genotype with that of the *B. suis* biovar 1 vaccine strain S2 according to the MLVA cluster analysis.

## Discussion

Since the establishment of sentinel surveillance points, a collection of animal and human isolates have been collected and analyzed. Analysis of the molecular characteristics of isolates recovered from the nationwide surveillance can reflect the epidemic features of brucellosis. *B. melitensis* has been shown to be the leading cause of brucellosis in the different epidemic regions of China [5,7]. The Inner Mongolia Autonomous Region is the most severely affected province in China, suffering from *B. melitensis* as the dominant species, as well as *B. abortus* and *B. suis* as the next most prevalent species [26].

The *B. melitensis* isolates displayed a greater diversity than *B. abortus* when the MLVA11 markers are considered. It is likely that the diversity of *B. melitensis* isolates in China is related with the high frequency of infected sheep and their subsequent movement throughout China. MLVA8 genotypes 42, 43, 58 and 63 were detected in humans, MLVA8 genotypes 42 and 63 were detected in domestic animals. The two *B. melitensis* MLVA8 genotype 47 isolates recovered from *Pseudois nayaur* (a wild animal) in Qinghai, were exceptions to this finding. However, this is the first report of the ‘Americas’ group in China. Whole genome sequencing of isolates from this lineage might provide information about host adaptation and microevolution. MLVA8 genotype 42, ‘East Mediterranean’ group (humans and animals isolates), as we have shown, is widely distributed throughout China, and has previously been reported to be predominant in Turkey, Portugal and Spain [19,24,27,28]. In Zhejiang province, an area with low prevalence of brucellosis, applying MLVA added valuable information to the epidemiological investigation and could be used as a tool to follow the spread and control of the disease. Additionally, the cluster analysis of *B. melitensis* forms a heterogeneous group including all three biovars (biovars 1, 2 and 3). Neither MLVA nor multilocus sequence typing distinguished the *B. melitensis* biovars [19,29,30]. This indicates that variable number tandem-repeat loci and the single-nucleotide polymorphisms which provide congruent data evolve independently of the putative genetic determinants for these biovars [21].

Based upon the year of *B. abortus* isolation, it is evident that isolates designated as genotype 36 (by MLVA panel 1) appear to have persisted in China since the 1980s and maybe associated with spread of isolates from northern to southern China; more genotyping data will need to be collected to re-enforce these observations. Although the number of human *B. abortus* isolates included in the study were modest, comparison of the genotypes of humans and animal isolates are very important to prevent the expansion and achieve the eradication of the disease particularly in areas with low prevalence of brucellosis.

In this study, the phenomenon of host shift is uncommon between *B. suis* (swine) and the accessory hosts (eg, sheep, cattle, and spotted deer). Considering MLVA results on S2 strain, we conclude that S2 is genetically very stable when the strain was passaged 40 times *in vitro*. We therefore believe that MLVA typing could be an essential assay to guarantee the quality and stability of the live attenuated vaccine. Detection of field isolates with the same MLVA pattern as the *B. suis* vaccine strain S2 raises the concern of the origin of the field isolates. The fact that strains 9, 210 and 218 were grouped together strongly suggests that the *B. suis* vaccine strain S2 has resulted in cross infection between these three animal species. This is not unexpected since the vaccine strain S2 was widely used in China. Results obtained by Garcia-Yoldi et al. also support that the *B. melitensis* vaccine strain Rev. 1 group as assayed by MLVA is genetically very homogeneous [25]. These findings suggest that the MLVA assay is useful for trace-back investigations during an epidemiological investigation.

The results presented in this study have highlighted some of the potential hazards associated with using the S2 vaccine in national control programs. Implementation of whole-flock vaccination procedures, including vaccination of pregnant animals, has led to outbreaks of abortions in several intensively managed flocks and isolation of the strain from the milk of the aborting animals [31]. Detailed analysis of relevant data must be reviewed by policy makers in order to revise current national vaccine standard guidelines/regulations. To date, human infection with the vaccine strain S2 in China has not been reported. However, in previous reports, isolates were only biotyped using conventional methods and no direct molecular linkage was shown between isolates and the commercially used S2 vaccine strain.

### Over the last 20 years, the geographic distribution of brucellosis in China has been

changing from pastoral areas to periurban and urban areas, according to information extracted from the Chinese National Notifiable Disease Surveillance System. It is worth noting that brucellosis has become endemic in southern provinces in China; these provinces tend to have higher gross domestic revenue and are more developed than other areas in China. On the other hand, alterations in socioeconomic and political systems, increasing animal trade and a decreasing awareness by practitioners and public health authorities led to the re-emergence of new endemic foci. Our results indicate that MLVA typing could be an essential assay to determine strain relatedness and trace-back investigation in endemic or non-endemic regions of brucellosis.

## Conclusions

From the collection of isolates analyzed in this study we conclude that MLVA confirmed the epidemiological linkage in outbreak and trace-back investigations. MLVA appears to be an important molecular genotyping tool that will enable improved surveillance for brucellosis and assist investigators in assessing what approaches will inhibit or reduce transmission of the diseases among animals as well as humans [32,33].

## Material and Methods

### Ethics Statement

This study is a retrospective investigation of our institute strain collection with modern typing methods. The study does not involve the collection or reporting of patient data, and no patient intervention occurred with the obtained results.

### Bacterial isolates and DNA preparation

82 *B. melitensis* isolates from 1960s to 2010s (78 recovered from animals, 4 isolated from an outbreak in humans), 70 *B. abortus* isolates from 1980s to 2010s (66 recovered from animals and 4 isolated humans) and 16 *B. suis* biovar 1 isolates from 1960s to 2010s (12 recovered from animals and 4 from humans) were examined. The *B. suis* biovar 1 vaccine strain S2 and the reference strain 1330 (ATCC 23444) were included. All field isolates were stored in department of brucellosis, China CDC. Bacterial isolates were cultured on trypticase soy agar containing 5% sheep’s blood (BD Diagnostic Systems, China Ltd., Beijing, China) at 37°C for 48 h. All isolates were identified as *Brucella* species on the basis of classical identification procedures: CO_2_ requirement, H_2_S production, inhibition of growth by basic fuchsin and thionin, agglutination with monospecific antisera, and phage typing [34]. Total genomic DNA was extracted using the DNeasy Blood & Tissue Kit (Qiagen China Ltd., Beijing, China) following the manufacturer’s protocol for extraction of genomic DNA from Gram-negative bacteria. Species-level identification was undertaken by the AMOS-PCR assay [35].

### MLVA-16 genotyping scheme

MLVA was performed as described previously with the following modifications [6]. The 16 primer pairs were divided into three groups: panel 1 (8 loci including bruce06, bruce08, bruce11, bruce12, bruce42, bruce43, bruce45, and bruce55), panel 2A (3 loci including bruce18, bruce19, bruce21), and panel 2B (5 loci including bruce04, bruce07, bruce09, bruce16, and bruce30). Forward primers of panel 2 loci were labeled with one of four 5’-fluorescent labels (6-FAM, ROX, HEX, or TAMRA). These primers were obtained from Shenggong Biosciences, Inc., (Shanghai, China). PCR conditions were as follows: initial denaturation at 94°C for 3 min followed by 30 cycles of 94°C for 30 s, 60°C for 30 s and 72°C for 50 s. The panel 1 loci amplicons were analyzed by gel electrophoresis as described previously [6,19]. Agarose gels were then normalized and band sizes were estimated using BioNumerics version 5.1 software (Applied Maths, Belgium). PCR products of panel 2 loci were denatured and resolved by capillary electrophoresis on an ABI Prism 3130 automated fluorescent capillary DNA sequencer (Applied Biosystems). Fragments were sized by comparison to a ROX (carboxy-X-rhodamine)-labeled molecular ladder (MapMaker 1000; BioVentures Inc., Murfreesboro, TN, USA) with GeneMapper, version 4.0 software (Applied Biosystems). Both the band size estimates and the fragments size were then converted to repeat units by following the published allele numbering system (Le Flèche et al. 2006 version 3.3: http://mlva.u-psud.fr *Brucella* support web site for MLVA typing).

### Stability of the MLVA loci

MLVA loci stability was investigated after *in vitro* passage experiments. The *Brucella* vaccine strain S2 was inoculated on trypticase soy agar containing 5% sheep’s blood (BD Diagnostic Systems) at 37°C for 48 h. A single bacterial colony was plated to fresh medium 40 times at approximately 2-days intervals and subjected to MLVA genotyping at each interval. Isolate MLVA profiles were compared with that obtained from the original seed stock to determine the stability of tandem repeat patterns at each of the 16 loci.

### Analysis of MLVA data

All data were analyzed using BioNumerics version 5.1 software (Applied Maths, Belgium). Cluster analysis was based on the categorical coefficient and unweighted pair group method using arithmetic averages (UPGMA) method. Polymorphisms at each loci were quantified using the Hunter & Gaston diversity index (HGDI) [36] via the online tool V-DICE available at the HPA website (http://www.hpa-bioinformatics.org.uk/cgi-bin/DICI/DICI.pl).

Resultant genotypes were compared using the web-based Brucella2012 MLVA database (http://mlva.u-psud.fr/). The genotyping data can be found in the supplementary file (Table S1).

## Supporting Information

Table S1 **MLVA-16 genotypes for all *Brucella* isolates.** The coding convention for in silico MLVA typing of reference strain 16M accession number NC_003317.1 and NC_003318.1.
(DOCX)
