# Supplementary material for: MLVA Genotyping of Brucella melitensis and Brucella abortus Isolates from Different Animal Species and Humans and Identification of Brucella suis Vaccine Strain S2 from Cattle in China
Source: PLoS One. 2013 Oct 4;8(10):e76332. doi: 10.1371/journal.pone.0076332 (PMC3790686; doi:10.1371/journal.pone.0076332)
Supplement: Table S1 — MLVA-16 genotypes for all Brucella isolates. The coding convention for in silico MLVA typing of reference strain 16M accession number NC_003317.1 and NC_003318.1. (DOCX) [file pone.0076332.s001.docx]

**Supporting information**

**Table S1 MLVA-16 genotypes for all *Brucella* isolates**

the coding convention for in silico MLVA typing of reference strain 16M accession number NC_003317.1 and NC_003318.1.
